# Liposomal nanoparticles as a drug delivery system for improved treatment of multiple myeloma

**DOI:** 10.1039/d5ra09823h

**Published:** 2026-03-13

**Authors:** Nemany A. N. Hanafy, Safacan Kolemen

**Affiliations:** a Koç University Research Center for Translational Medicine (KUTTAM) 34450 Istanbul Turkiye skolemen@ku.edu.tr nhanafy@ku.edu.tr; b Group of Bionanotechnology and Molecular Cell Biology, Nanomedicine Department, Institute of Nanoscience and Nanotechnology, Kafrelsheikh University 33516 Kafrelsheikh Egypt; c Department of Chemistry, Koç University 34450 Istanbul Turkiye

## Abstract

Multiple myeloma (MM) remains a challenging type of hematological cancer, characterized by the growth and accumulation of clonal plasma cells within the bone marrow. Current traditional therapies, mainly conventional chemotherapeutics, demonstrate limited therapeutic capacity due to considerable side effects and drug resistance. In this direction, liposomal nanotherapeutics have emerged as a promising new strategy in cancer therapy, holding great potential to address the chemical and biological constraints of existing anticancer treatments. This review presents a summary of the latest developments in the application of targeted liposomal nanoparticles as a drug carrier system for the management of MM. Furthermore, it explores the challenges linked to creating efficient drug delivery systems, targeting methods, and the processes involved in controlled drug release.

## Abstract 2

This review summarizes recent advances in liposomal nanoparticle-based drug delivery systems aimed at improving therapeutic outcomes in multiple myeloma.

## Graphical abstract

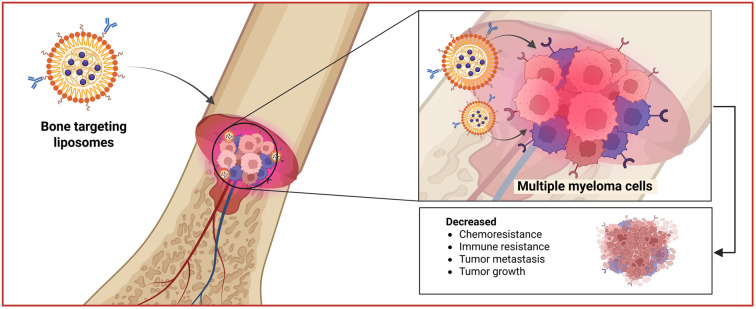

## 1. Introduction

Multiple Myeloma (MM) is the second most prevalent hematological malignancy, representing more than 13% of blood cancer cases.^1^ 63% of MM patients are over 65 years old, and the average age of diagnosis is 69.^2^ MM has a yearly mortality rate of 2.9 for every 100 000 individuals, based on surveillance, epidemiology, and results (SEER) statistics. MM occurs more frequently in males than in females, and it is twice as widespread among those of African descent.^3^ As per the American Cancer Society, it is anticipated that around 36 110 new cases of MM will be diagnosed in the US in the coming year (20 030 in men and 16 080 in women), with around 12 030 deaths expected (6540 in males and 5490 in females).^4^ In MM, there is a significant rise in plasma cells, which are derived from B cells that have experienced somatic hypermutation due to antigen exposure. In individuals with good health, this mechanism produces enduring plasma cells that are crucial for maintaining the immune system's production of polyclonal antibodies. Conversely, clonal plasma cells generate an excessive number of monoclonal immunoglobulins or light chains in MM.^5^ The characteristic clinical signs of MM, including anemia, bone abnormalities, immune suppression, elevated calcium levels, and kidney impairment, are mainly ascribed to the buildup of clonal plasma cells in the bone marrow and the generation of monoclonal protein.^6^ The cytokines that govern the persistence of MM cells are generated due to the interplay between tumour cells and bone marrow stromal cells. Among the various cytokines that affect the differentiation and proliferation of MM cells, interleukin-6 (IL-6) is the key growth factor, as it inhibits apoptosis induced by various medications. Furthermore, it significantly contributes to the development of clinical treatment resistance.^7^

A combination of genetic and environmental factors is thought to underlie the pathophysiology of multiple myeloma, leading to the production of high levels of monoclonal immunoglobulins and the malignant transformation of plasma cells.^8^ Protease inhibitors, immunomodulatory medications (IMiDs), monoclonal antibodies, and histone deacetylase inhibitors are instances of recent advancements in treatment that have significantly enhanced therapeutic results. Rates of relapse and mortality remain elevated, which highlights the importance of continuous investigation into innovative treatment methods to enhance patients' long-term survival.^9^ Despite progress in therapy, the average survival for MM stays within the range of 4.4 to 7.1 years. The condition often recurs after apparent total remission, mainly because of the emergence of drug resistance.^10^ The illness advances through three phases: an active stage characterized by a minor percentage (<1%) of growing plasmatic cells, a severe phase marked by extramedullary proliferation, and a dormant phase where tumour cells are mature plasma cells that do not proliferate. There are numerous opportunities for therapeutic intervention due to this layered transformation process and an extended median survival time.^11^ In this context, MM-targeted nanoparticles that carry therapeutic agents have garnered considerable interest as they offer a selective and highly effective treatment approach.

Recently, several reviews have explored various nanoparticle-based strategies as targeted approaches for the treatment of MM.^12,13^ However, this review specifically focuses on the application and modification of liposomes, as they hold significant potential as drug carriers in MM therapy due to their prolonged circulation time, storage stability, ease of functionalization, ability to co-load multiple chemotherapeutic agents, and efficient penetration into the bone marrow microenvironment. Accordingly, this review highlights the key features of liposomal nanotechnology, recent advances in chemotherapeutic-loaded liposomal nanoparticles, and the evolving design of liposomes functionalized with specific molecules or antibodies for targeted MM therapy. Liposome-encapsulated chemotherapeutic agents, administered either individually or in combination, are summarized with representative examples and detailed explanations for each approach. In addition, this review discusses numerous antibodies and targeting molecules recently used to functionalize liposomes for MM treatment, along with current limitations and future perspectives, providing a comprehensive and up-to-date overview.

## 2. Liposomal nanotechnology

Liposomes, engineered orbicular vesicles mainly made up of phospholipids and cholesterol, are organic substances found in living beings. Their biological properties make them an ideal vehicle for drug delivery due to their biocompatibility, biodegradability, and lack of immunogenic response.^14^ As a nanoscale medication delivery system, they have greatly improved the efficacy of standard treatments *via* regulated release, extended circulation durations, heightened tumour gathering, enhanced cell absorption, and reduced overall toxicity.^15^ Using liposomal nanoparticles as drug delivery carriers allows controlled release, targeted distribution within the body, and optimized pharmacokinetics, ensuring that treatments reach the tumor in the correct molar proportions.^16^ Liposome nanotechnology demonstrates potential in the realm of MM treatment by developing targeted therapeutic strategies that seek to enhance treatment efficacy while reducing drug-related toxicity.^17^ According to recent studies, specific elements of human serum liposomes are vital to the pathophysiology of MM.^18^

**Fig. 1:**
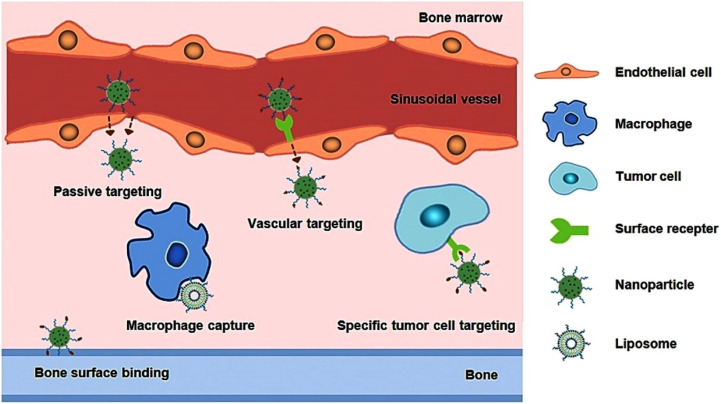
Schematic illustration of directed medication delivery methods to bone marrow. Adapted with permission from ref. 22. Copyright 2018 Elsevier.

When medications are given systemically for the management of bone marrow cancers such as leukemia and bone metastases, the availability of the drug is quite limited due to their rapid elimination, occasionally even before their ability to influence the intended diseased areas in the bone marrow.^19^ Most injectable medications either accumulate in other tissues or organs with high blood flow before arriving at the bone marrow or are removed by the body's metabolic/excretory processes. Consequently, to obtain a significant therapeutic impact within the bone marrow, drugs are consistently administered in large doses, which can lead to unavoidable systemic side effects.^20^ At this point, a comprehensive design approach is essential. In particular, the strategy must guarantee that therapeutic agents specifically accumulate in the lesion or tumour locations within the bone marrow, consequently improving effectiveness while reducing unintended side effects.^21^ At present, there are five main drug delivery approaches utilized in the management of tumors located in the bone marrow: precise targeting of tumor cells, capture mediated by macrophages, passive targeting, targeting of blood vessels, and attachment to bone surfaces (Fig. 1).^22^

Remarkably, individuals diagnosed with MM exhibit considerably reduced blood concentrations of acidic sphingomyelinase,^23^ and sphingolipids have been associated with the development of the disease.^24^ Additionally, the severity of MM could be linked to blood triglyceride concentrations.^25^ Despite these understandings, obstacles like small sample groups and restricted clinical information have led to varying results concerning the causal connection between MM and liposomal elements.^26^

Chemotherapy treatments often become ineffective in addressing bone metastasis due to the unavoidable progression of blood cancers and solid tumors that have spread to the bone in clinical settings. Administering intravenous anticancer medications in adequate therapeutic amounts in the bone marrow is a difficult task to inhibit tumour growth.^27^ Consequently, chemotherapy is generally given in high doses to attain sufficient therapeutic effectiveness in the cancerous bone marrow. Nonetheless, this method frequently leads to severe side effects, such as harm to healthy tissues and myelosuppression.^28^ On the other hand, the bone marrow microenvironment causes tumour cells, particularly cancer-initiating/stem cells, to develop resistance to chemotherapy and enables them to multiply, which can affect the relapse of the illness.^29^ Choosing the right medications for MM and effectively transporting it to the bone marrow are both challenging endeavors. Reduced blood flow and the bone marrow blood barrier, which limit the transport of small-molecule or nanoparticle-based medications to the bone marrow following systemic administration, are the main factors contributing to this issue.^30^ In addition, various methods have been proposed to employ aptamers, peptides, or antibody-altered nano-pharmaceuticals to specifically focus on the bone marrow; nevertheless, enhancing the effectiveness of delivery remains an important issue.^31^

**Fig. 2:**
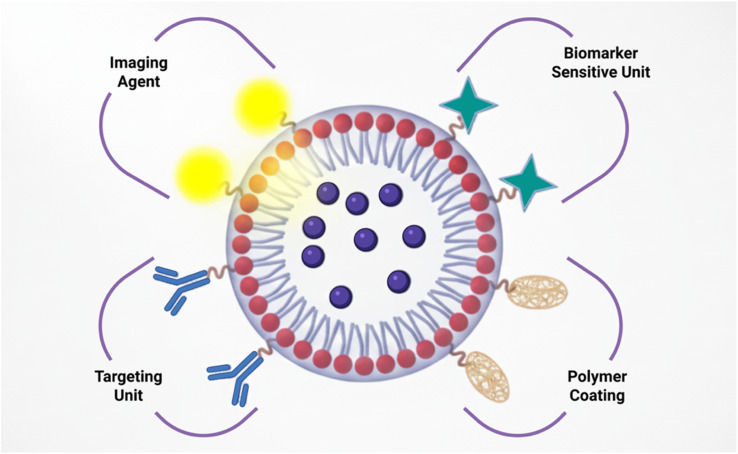
Possible uses of liposomes for treatment, imaging for diagnosis, and theranostics.

PEGylated liposomes are beneficial in the treatment of MM due to three well-recognized traits: (1) an inherent inclination for bone marrow (aside from the liver and spleen),^32^ where plasma cells gather, concentrate, and establish their nurturing environment; (2) a proven ability to aim at tumours generally through leveraging heightened vascular permeability resulting from inflammation and neovascularization and (3) PEG-liposomes demonstrate significant absorption by macrophages, a cell variety known for its critical role in controlling the advancement of various myeloid.^33^ PEG-liposomes may enable drug infiltration and retention within cells.^34^ In this instance, the ability of water-soluble polymers to induce interactions with cell membranes and thus promote the efficient transfer of encapsulated substances into the cytoplasm may be associated with the internal distribution of colloidal systems.^35^ PEG groups might additionally facilitate interactions between liposomes and cell membranes, leading to a process of interaction that occurs exclusively at the point where the membrane and lipid bilayer come into contact.^36^ These approaches can be applied to administer anticancer drugs through nanoparticle delivery mechanisms: passive targeting, which utilizes the pathophysiological conditions of the tumours, stimulated targeting, which causes the medication to be released once within the tumour employing triggered or inducible methods, or active targeting, which includes attaching high-affinity ligands to the exterior of nanoparticle delivery systems to aim for the tumour (Fig. 2).^37,38^

## 3. Chemotherapy medications enclosed within liposomes

### 3.1. Doxorubicin

Doxorubicin encapsulated PEGylated liposomal was the inaugural FDA-sanctioned nanoparticle delivery system for therapeutic application in MM. It has been integrated with additional anti-myeloma treatments, such as vincristine alongside dexamethasone or bortezomib. PEGylated liposomal doxorubicin (Tibotec Therapeutics) was administered on day 4 at 30 mg m^−2^ to individuals with relapsed or resistant MM, while bortezomib was administered on days 1, 4, 8, and 11 at doses of 0.90 to 1.50 mg m^−2^. When compared to bortezomib monotherapy (median time to progression (TTP) = 6.5 months), the duration until progression for the combination arm was significantly extended (median TTP = 9.3 months).^39^ PEGylated liposomal doxorubicin (40 mg m^−2^), vincristine (2.0 mg, day 1), and oral or intravenous dexamethasone (40 mg daily for 4 days) were given every 4 weeks for a minimum of 6 cycles, and/or for two cycles after achieving the best response, to individuals with newly diagnosed MM. With a total remission rate of 12%, the overall response rate was 88%. The median time to progression was 23.1 months, and progression-free survival rates were 42% and 23% at 2 and 3 years, respectively. These results are very encouraging regarding the clinical efficacy of NPs in treating MM; however, further follow-up data are necessary.^40^

### 3.2. Gemcitabine

Gemcitabine is an antimetabolite chemotherapeutic agent that inhibits DNA synthesis.^41^ It has been shown to induce apoptosis in MM cell lines and to arrest cells in the S phase of the cell cycle, independent of Bcl-2 and interleukin-6 (IL-6) expression levels. IL-6 is an important treatment alternative for myeloma patients; however, it did not offer any defense against gemcitabine-induced cell death when introduced to MM cell lines exposed to the medication. These results reinforced the use of gemcitabine as a therapeutic agent for treating MM in clinical research.^41^ Despite its efficacy, gemcitabine has a narrow therapeutic window and may cause significant adverse effects.^42^ Previous studies reported that liposomal formulations can inhibit P-glycoprotein-mediated drug efflux and that rhodamine-labeled liposomes are internalized by RPMI 8226 MM cells primarily through endocytosis.^43^ More recent investigations have demonstrated that the interaction between the vesicular structure and biological membranes may be improved by the charge of lipids and the linking of polymers, particularly PEG groups, at the liposome surface level. The assays for intracellular gemcitabine accumulation that confirmed and supported the confocal microscopy (CLSM) results provided further evidence of the improved intracellular transport enabled by the PEGylated liposomal vascular network.

The liposomal vascular system containing gemcitabine may serve as a suitable chemotherapy formulation for addressing MM, considering its critical nature as an untreatable blood cancer. *In vitro* studies have shown that this liposomal formulation offers notable advantages at the cellular level and may also enhance gemcitabine's pharmacokinetics and biodistribution. By enhancing anticancer efficacy and reducing the adverse effects of the drug, these factors could also contribute to improving the therapeutic index, thereby potentially increasing its application in clinical settings for the treatment of MM disease. As a result, the encouraging *in vitro* findings justify the necessity for additional *in vivo* studies.^36^

### 3.3. Mertansine

Mertansine, a microtubule inhibitor that is conjugated to a non-cleavable linker, 4-(*N*-maleimidomethyl) cyclohexane-1-carboxylate. Although it offers therapeutic promise for several cancer types, its use in clinical settings has been restricted due to inadequate pharmacokinetic characteristics, minimal solubility, and considerable dose-related toxic effects. To create liposomal formulations of mertansine, the method of dry film hydration and extrusion was utilized with a specified stoichiometric ratio of distearoylphosphatidylcholine (DSPC), PEGylated phospholipid featuring a saturated C18 stearoyl fatty acid, cholesterol, and a DM1 prodrug. The composition included 5 mol% of the mertansine prodrug and yielded an encapsulation efficiency of at least 90%. RME and CD138pep-lipid absorbed the structures. A polycarbonate membrane was utilized to create the liposomal nanoparticles for extrusion, yielding nanoparticles approximately 100 nm in size, which is optimal for passive targeting *via* the EPR effect. The study hypothesizes that the prodrug's optimized chemical structure enables efficient loading into nanostructures and controlled release following endocytosis by cancer cells, ultimately resulting in significant tumor growth inhibition. Four functional linkers; amide, ester, disulfide, and phosphodiester, were evaluated for the creation of DM1-pro to examine this hypothesis; they were labeled Prodrug-1, -2, -3, and -4, correspondingly. The targeting technique was based on the CD138 peptide–lipid conjugate, which was included at a concentration ranging from 0.1% to 1% to address MM cells that exhibited heightened CD-138 levels. It was demonstrated that the oligolysine composition of the peptide led to a rise in the zeta potential as the peptide concentration increased. Thioether linkers were utilized to attach the mertansine prodrug molecules to the lipid tails of the nanoparticles, improving their durability against premature hydrolysis and facilitating the regulated release of the drug into the cells.^44^ High performance liquid chromatography (HPLC) was used to evaluate the release profile of encapsulated mertansine in comparison with its non-encapsulated form. The results confirmed sustained release of mertansine from liposome-loaded nanoparticles for up to 48 h under both physiological (pH 7.4) and acidic (pH 4.8) conditions. This sustained retention underscores the potential of liposomal nanoparticles in MM therapy by preventing premature drug leakage before reaching the target site.^44^

### 3.4. Bortezomib

Bortezomib (BTZ), the pioneering proteasome inhibitor approved by the FDA for managing various stages of MM, exerts its anticancer properties *via* numerous mechanisms, such as blocking the 26S proteasome, stopping the degradation of p53, activating caspases, promoting the production of reactive oxygen species (ROS), phosphorylating Bcl-2, increasing NOXA levels, and altering NF-κB communication, and suppression of blood vessel formation.^45^

Over time, resistance to BTZ treatment remains an unavoidable challenge for all MM patients. Both tumor-inherent and tumor-external factors are essential for the operation of BTZ resistance mechanisms. Recent studies, for example, have elucidated how mutations or increased expression of proteasome subunits, particularly β5, lead to BTZ resistance (39, 40), while additional research has highlighted the significant influence of host-mediated pro-tumorigenic responses. Encasing BTZ in liposomes with CXCR4 antagonist AMD3100 (Mozobil), a moiety aimed at the bone, represents a potentially impactful therapeutic strategy for MM and various other bone-associated disorders.^32^

**Fig. 3:**
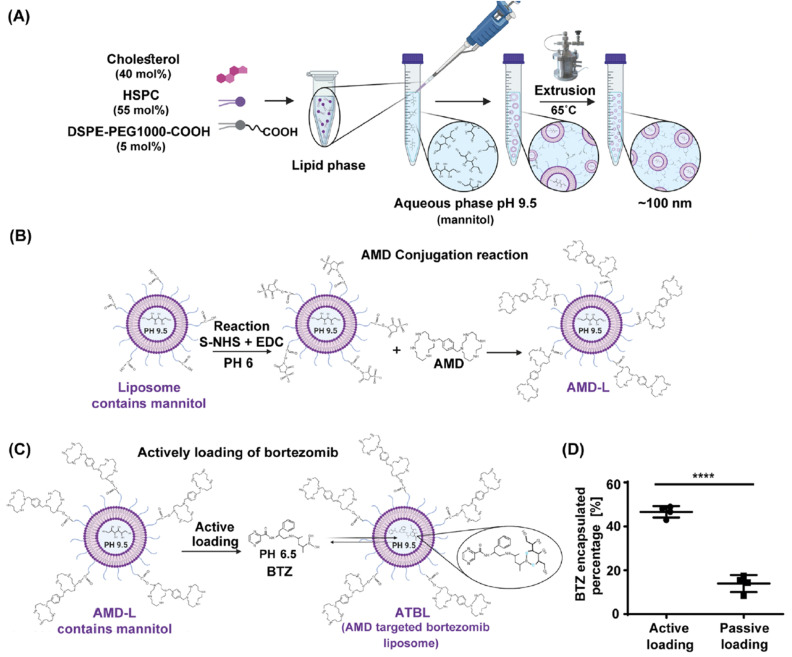
Synthesis of AMD-conjugated, BTZ-encapsulated liposomes for targeting MM cells within the bone marrow. (A) A representation of the technique for producing liposomes *via* the ethanol injection method into a water phase containing mannitol and acetic acid. At a pH of 9.5, succeeded by extrusion to ensure a size of approximately 100 nm. (B) A schematic illustration utilizing the S-NHS response and EDC to satisfy AMD conjugation to the liposomes. (C) Utilization of pH and concentration gradients to show the active loading of BTZ into liposomes. (D) A comparison between active and passive loading for BTZ encapsulation into liposomes (*n* = 4). Adapted with permission under a Creative Commons CC BY License from ref. 32. Copyright 2025 American Chemical Society.

The encapsulation efficacy of liposomal bortezomib nanoparticles was recorded at 80%, and their dimensions were determined to be approximately 100 nm with impressive consistency. Proteasome blockage, cell death, and cell viability were analyzed *in vitro*. Liposomal bortezomib nanoparticles (NPs) caused cell damage, initiated apoptosis, and suppressed proteasome function in MM cells. In *in vivo* studies, SCID (severe combined immunodeficiency) mice were implanted with MM cells subcutaneously and received intravenous treatment with either the unbound drug or liposomal bortezomib NPs on days 1 and 4, at a dosage equivalent to 1 mg kg^−1^ of bortezomib. Tumor progression and overall toxicity were then evaluated.^46^ These results demonstrated that liposomal bortezomib nanoparticles were successful in reducing systemic side effects, as evidenced by less significant body weight loss. On the seventh day, the mice receiving the free drug experienced a loss of over 20% of their body weight and were in a critical state, leading to their euthanasia. In the case of liposomal bortezomib nanoparticles, the 2-week testing duration led to a decrease in body weight of under 10%. Distribution tests ought to have been conducted to verify the positioning of the NPs, even with the reduced side effects (Fig. 3).^32,47^ The amount of BTZ release was determined by dividing the total encapsulated BTZ concentration by the free BTZ. Accordingly, under conditions of 50% serum at 37 °C, approximately 40% of BTZ was released from the liposomes over a period of 10 days.

### 3.5. Anti-estrogen

RU 58,668 is a new steroidal anti-estrogen. In MCF-7 cells stimulated by estradiol or by external or internal growth factors, RU 58,668 displayed a significant antiproliferative impact (IC50, 0.01 nM). Moreover, it inhibited the growth of the insulin-stimulated T47D cell line. In the absence of any agonistic effects, RU 58,668 triggered total anti-uterotrophic activity in mice or rats in a living organism.^48^ Anti-estrogens have been integrated into liposomes to inhibit their overall distribution after oral intake and to reduce their buildup in unintended tissues. The liposomes exhibited an encapsulation efficacy exceeding 90% and measured approximately 100 nm in size. In an MM xenograft model, the loaded liposomes were administered intravenously at a dosage of 12 mg anti-estrogen per kg per week. In contrast to empty liposomes or unbound anti-estrogen, the loaded liposomes halted the tumour growth. Consequently, the administration of anti-estrogen medication improves their capacity to suppress the development of tumors that express estrogen receptors, rendering it especially potent for addressing estrogen-dependent breast cancer. Additionally, it represents an innovative and efficient approach to treating MM.^49^

### 3.6. Homoharringtonine

Homoharringtonine (HHT), which was extracted from the *Cephalotaxus harringtonia* tree, has been widely utilized in traditional Chinese medicine to address various hematologic cancers. The FDA has approved HHT to be used in the treatment of individuals with acute and chronic myeloid lymphoma. However, HHT can lead to unforeseen gastrointestinal side effects, limiting its extensive therapeutic use.^50^ Anticancer drugs have gained effectiveness and reduced toxicity thanks to the rising application of liposome encapsulation, which alters the pharmacokinetics and distribution of the drugs in a living organism. The long-circulating, dose-independent liposome formulations with PEG on the liposome exterior have been authorized for the therapy of different tumours, according to mounting evidence. Recent *in vitro* studies utilizing PEGylated long-circulating liposomes encapsulating homoharringtonine have shown substantial suppression of MM cancer cells or MM cancer stem cells.^51^

### 3.7. Dexamethasone

**Fig. 4:**
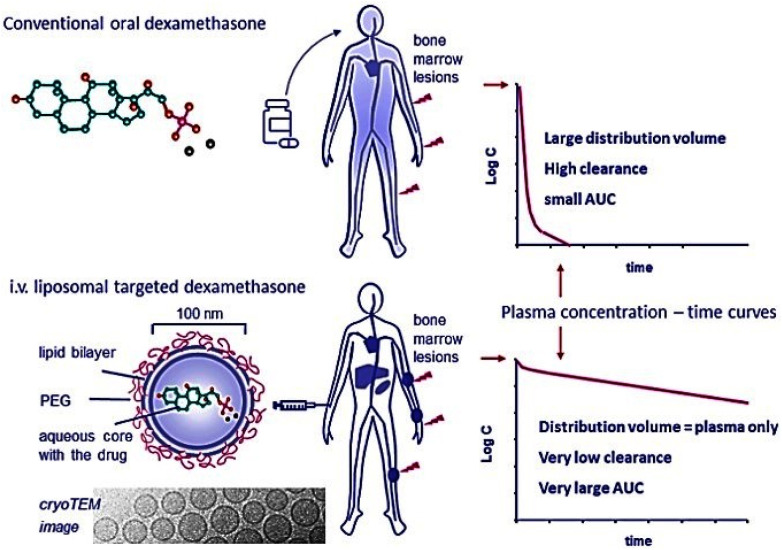
Schematic illustration of the difference between intravenous PEG-liposomal dexamethasone administration and conventional oral dexamethasone treatment, emphasizing especially the significant discrepancies in pharmacokinetic profiles and site-specific accumulation after liposomal therapy. Adapted with permission from ref. 38. Copyright 2023 Springer Nature.

Dexamethasone is utilized in medical environments to manage MM and other cancers driven by inflammation.^52^ Dexamethasone is commonly used in combination with various chemotherapeutic agents, such as immune modulators (thalidomide and lenalidomide), proteasome inhibitors (bortezomib and carfilzomib), cyclophosphamide, or melphalan, in treatment protocols for individuals with MM.^53^ Clinical outcomes in MM patients have shown improvement when dexamethasone is incorporated into treatment plans.^54^ Unintended side effects of glucocorticoids encompass significant systemic immunosuppression, potentially leading to opportunistic infections that, if not addressed, can be deadly.^55^ Osteoporosis, osteonecrosis, muscle disease, stunted growth in children, high blood pressure, swift weight increase, redistribution of fat, diabetes, elevated triglycerides, hypercholesterolemia, adrenal insufficiency, skin thickening, glaucoma, cataracts, peptic ulcer disease, delayed wound healing, and electrolyte disturbance are further adverse effects of GCs.^56^ In addition, the pharmacokinetic characteristics of glucocorticoids are suboptimal, marked by rapid elimination and a large distribution volume after administration. A significant and consistent dosage is required.^57^ Liposomal encapsulation of dexamethasone has been demonstrated to improve localized levels in inflamed tissues while minimizing exposure to vulnerable organs. These results indicate that liposomal encapsulation can augment the pharmacokinetic and pharmacodynamic properties of dexamethasone, thereby boosting its therapeutic index. A reduced dosage of dexamethasone could be utilized in clinical applications with existing combination therapies by using liposomal formulations, potentially improving treatment outcomes and decreasing dosage-related side effects (Fig. 4).^38,58^*In vitro* release kinetics of the liposomal formulation of dexamethasone was also assessed.^57^ The formulation was found to be stable *in vitro*, releasing just 5% of the medication that was encapsulated after two weeks at 37 °C.

### 3.8. Carfilzomib

The FDA has approved carfilzomib, a second-generation proteasome inhibitor that originates from epoxomicin, for the treatment of MM. Carfilzomib interferes with proteasome-dependent mechanisms, initiates cellular stress responses, and triggers apoptosis by permanently attaching to the chymotrypsin catalytic site of the 20S proteasome.^59^ While carfilzomib is a successful therapeutic option, it comes with adverse effects, such as thrombocytopenia occurring in 23% of individuals and grade 3/4 anemia in 22% of individuals.^60^ The enhanced EPR effect enables nanoparticles ranging from 20 to 200 nm in size to preferentially cluster and aim for the tumour site.^61^ Liposomal carfilzomib nanoparticles effectively target MM cells and show improved effectiveness *in vivo*. In comparison to free carfilzomib, both non-targeted carfilzomib and targeted carfilzomib showed considerable tumour growth suppression *in vivo*, reduced systemic toxic effects, and increased cytotoxic effects *in vitro*.^47,62^ The release kinetics of carfilzomib from liposomes were evaluated using LEP and HPLC analyses. The release profile demonstrated that the nanoparticles effectively retained carfilzomib for up to 72 h at both room temperature and physiological temperature, as well as across a pH range of 7.4 to 5.5. Free carfilzomib was used as a control in these experiments. Such sustained drug retention is advantageous in nanoparticle-based drug delivery, as it ensures retention of the therapeutic agent within the carrier until it reaches the tumor site.^62^

Table 1 summarizes the chemotherapy drugs loaded in different liposomes and tested cell lines along with key outcomes obtained from the chemotherapeutics encapsulated in functionalized liposomes.

**Table 1:** Chemotherapeutics encapsulated in functionalized liposomes to treat MM and their key outcomes

Liposomal functionalization	Chemotherapeutics	Cell lines	Key outcomes	Ref.
Pegylated liposome	Doxorubicin	Patients with MM	Treatment of patients who are restricted from corticosteroid-containing therapy	40
Pegylated liposome	Gemcitabine	RPMI 8226	Improved cellular uptake, gemcitabine pharmacokinetics, and biodistribution	43
Thioether functionalized pegylated liposome	Mertansine	NCI-H929 MM cells	High level of anti-tumor activity while lowering the systemic toxicity of clinically authorized treatments	44
Pegylated liposome	Bortezomib	CAG, U266, and RPMI8226 (RPMI) human MM cell lines	Bone-targeting moiety in the form of AMD3100	32 and 47
Pegylated liposome	RU 58,668	LP-1, OPM-2, NCI-H929, RPMI 8226, U266 and Karpas 620	A new therapeutic strategy for anti-estrogen administration	49
Pegylated liposome	Homoharringtonine	RPMI 8226	Improved bioavailability in preventing cancer stem cells of MM development, while reducing chemotherapeutic side effects	51
Pegylated liposome	Dexamethasone	MM.1S	Enhanced pharmacokinetic and pharmacodynamic characteristics of dexamethasone, improved therapeutic index	57
PEGylated liposomal	Carfilzomib	MM.1S and NCI-H929 cells	Improved therapeutic index and greatly increased patient outcomes	62

## 4. Combinatorial therapies

Combinatorial treatments continue to be crucial in the treatment of MM.^63^ Formulations that supply the drugs directly to the tumor site in their optimal synergistic proportions are essential for enhancing the efficacy of combination therapies. While existing combination therapies are effective, managing the drug ratio at the tumor location is very challenging because of variations in the pharmacokinetics, biodistribution, and metabolism of the individual drugs.^64^ Therefore, the therapeutic outcome could be greatly influenced by utilizing nanoparticles as carriers for drug delivery in combination therapies.

**Fig. 5:**
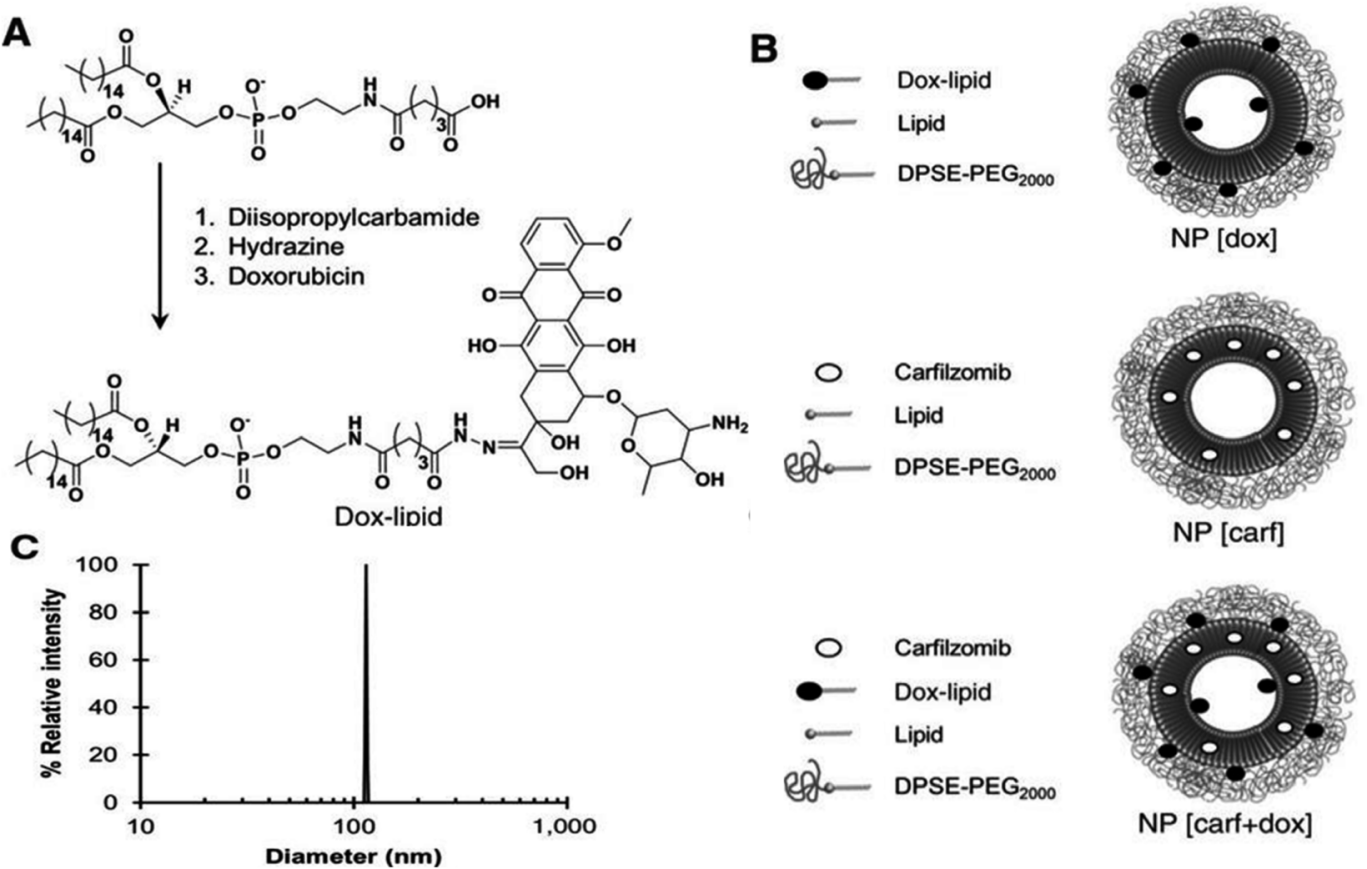
(A) Schematic illustration showing the conjugation of doxorubicin to DPPE-GA *via* a labile hydrazone bond. (B) Representative structures of the dual drug-loaded liposome NP[carf + dox] (bottom) and the single drug-loaded liposomes NP[carf] (middle) and NP[dox] (top). (C) Dynamic light scattering (DLS) measurements of the various liposomal nanoparticles, showing an average particle diameter of approximately 115 nm for NP[carf], NP[dox], and NP[carf + dox]. Adapted with permission under a Creative Commons CC BY License from ref. 65. Copyright 2016 American Association for Cancer Research.

Consequently, Bilgicer and colleagues demonstrated, for the first time, the combination of doxorubicin and carfilzomib and their incorporation into nanoparticles for increased therapeutic effectiveness. To transport carfilzomib and doxorubicin to cancer cells at their optimal synergistic proportion for improved therapeutic effect, they encapsulated carfilzomib and doxorubicin. Their findings exhibited that encapsulated carfilzomib and doxorubicin proved to be more potent both *in vitro* and *in vivo* compared to the unbound drug combination. The research revealed the collaborative effects of carfilzomib and doxorubicin, along with the potential for their integration to form nanoparticles towards enhancing treatment results. When assessed as a whole, this research displays the medicinal promise of these initial-generation liposomal nanoparticles infused with doxorubicin and carfilzomib, alongside the preclinical rationale for the clinical advancement and evaluation of encapsulated carfilzomib and doxorubicin for better patient outcomes in MM and the preclinical rationale for the clinical advancement and evaluation of encapsulated carfilzomib and doxorubicin for better patient outcomes in MM.^65^ A doxorubicin–lipid prodrug by conjugating doxorubicin to the polar head group of a DPPE lipid *via* a hydrolyzable hydrazone bond was developed, enabling efficient incorporation of doxorubicin into the nanoparticles. The gradual hydrolysis of this labile bond allows for controlled release of doxorubicin from the nanoparticle surface. Carfilzomib was subsequently loaded into the liposomes by co-formulation with the other lipids prior to film formation at a molar ratio of 94 : 5 : 1 (DSPC : DSPE-PEG2000 : carfilzomib). Owing to its hydrophobic nature, carfilzomib was efficiently embedded within the lipid bilayer of the liposomes. Based on these findings, the combination of pegylated liposomal doxorubicin with bortezomib has been proposed as a potential standard-of-care regimen for this subset of MM patients, particularly those with high-risk disease (Fig. 5).^65^

## 5. Chimeric antigen receptor

Chimeric antigen receptor (CAR) T-cell treatment has recently attained significant clinical responses and improved the therapeutic results in relapsed/refractory acute lymphoblastic leukemia and diffuse large B-cell lymphoma, resulting in their FDA endorsement. Nevertheless, existing cellular therapies, such as CAR-T treatment, necessitate intricate patient-specific protocols for the development and proliferation of effector cells, which are both resource-demanding and financially inefficient.^66^ Nonetheless, the design and expansion of effector cells for modern cellular therapies, including CAR-T therapy, require complex personalized protocols that are both costly and labor-intensive.^62^

## 6. Proactive targeting methods for multiple myeloma

### 6.1. Very late antigen-4

A vital adhesion molecule that is highly expressed in the majority of MM instances, Very Late Antigen-4 (VLA-4; known as α4β1 integrin), serves as a surface receptor that can be utilized therapeutically for targeting MM.^67^ The vascular cell adhesion molecule-1 (VCAM-1) and fibronectin are two well-known ligands that have QIDS (Gln-Ile-Asp-Ser) and ILDV (Ile-Leu-Asp-Val) sequences that are identified by the non-covalent, heterodimeric, transmembrane receptor VLA-4. Individuals with active MM displayed the greatest presence of plasma cell adhesion molecules in human MM specimens.^68^ Moreover, VLA-4 has been associated with enhancing the function of bone-resorbing osteoclasts in MM by promoting the secretion of osteoclast-activating factors such as MIP-1α and MIP-1β.^69^ These findings suggest that VLA-4 is a marker for MM, which is correlated with the migration of myeloma cells. Utilizing the mouse bone marrow stromal cell line ST2, which produces VCAM-1, alongside the α4β1 (VLA-4) positive murine myeloma cell line 5TGM1, Michigami *et al.* illustrated that myeloma cells engaging with stromal cells through α4β1-integrin/VCAM-1 leads to osteoclastogenic activity, signifying that the existence of stromal cells creates an environment for myeloma cells to exclusively inhabit the bone marrow.^70^

Increased expression of VLA-4 has been detected in MM cells and the adjacent stroma. Furthermore, the stroma of myeloma bone marrow contains a high concentration of VLA-4 ligands, including vascular cell adhesion molecule-1 and fibronectin.^67^ In MM, inside-out signaling leads to a VLA-4 structural alteration to an activated form, initiating high-affinity ligand attachment, which subsequently activates additional intracellular signaling (outside-in signaling).^71^ The conformational activation of VLA-4 is associated with increased cell movement, growth, and resistance to medications.^72^ The application of VLA-4-targeted nanoparticles has been investigated to improve the targeting of VLA-4-expressing MM cells. Nonetheless, the expression of VLA-4 in human myeloma cells is highly variable, with some MM cells showing minimal expression; additionally, VLA-4 is found in various other non-myeloma cell types.^73^ Evaluation of myeloma cell surface biomarker expression through a personalized medicine strategy will be necessary in the context of clinical methodologies.^74^

**Fig. 6:**
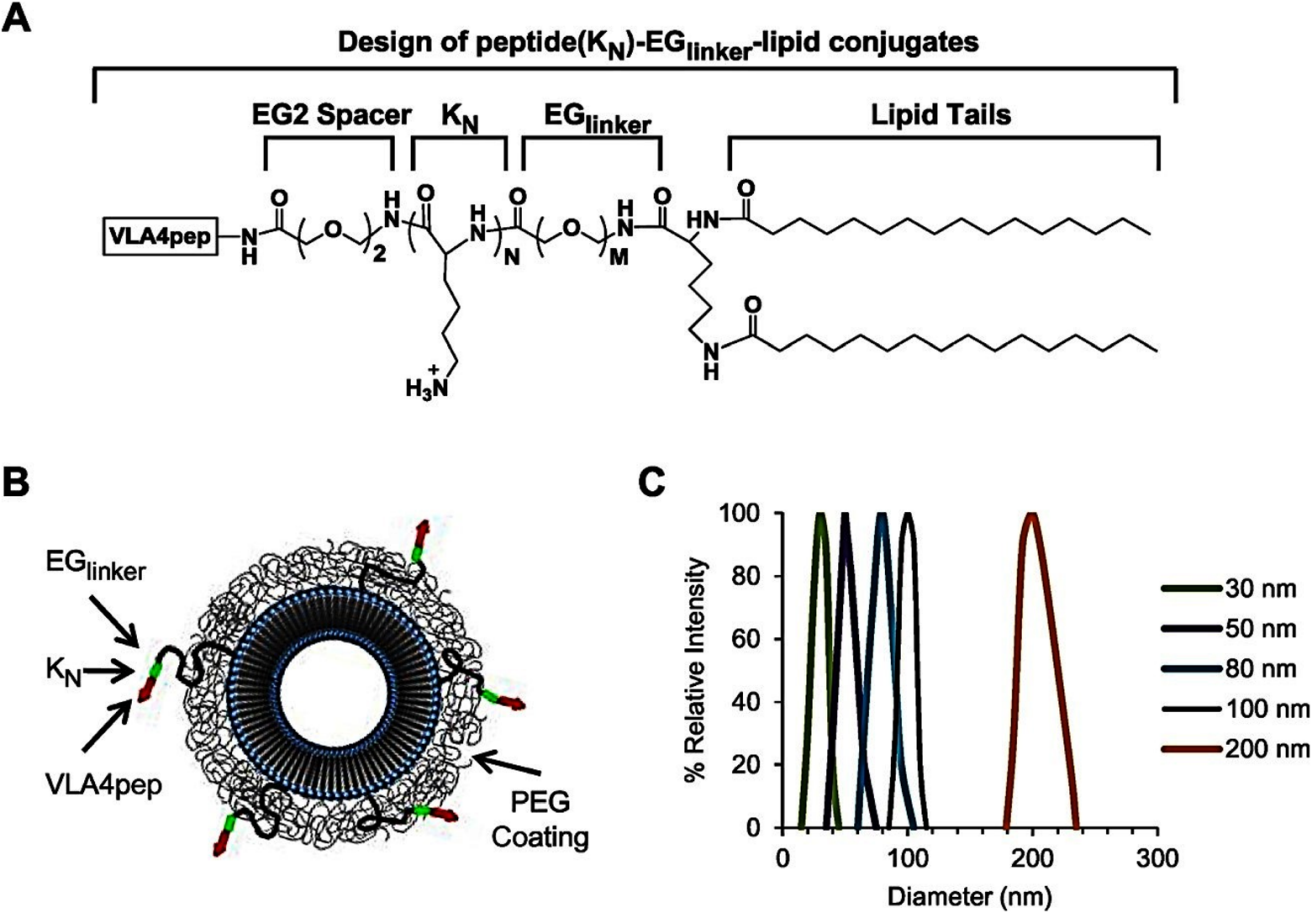
Schematic description of nanoparticles associated with peptides. (A) Lengths of EG peptide-linkers, including EG6, EG12, EG18, EG24, EG30, EG36, EG45, and EG72, and peptide (KN)–EGlinker–lipid combinations with differing oligolysine (KN) levels. (B) Schematic of peptide-targeted liposomes. (C) Examination of dynamic light scattering from nanoparticles of varying sizes. Adapted with permission from ref. 75. Copyright 2019 Elsevier.

Liposomes were modified with a cyclic VLA-4 antagonist peptide (VLA4pep) that was adjusted using EG peptide-linkers of different lengths, along with a brief oligomycin sequence to enhance hydrophilicity over various peptide densities and liposome dimensions, to assess how these factors influence the biological outcomes (Fig. 6).^75,76^

### 6.2. CXCR4

The well-preserved architecture of the seven-transmembrane G protein-coupled receptor CXCR4 consists of 352 amino acid residues, featuring three extracellular and intracellular loops, seven transmembrane helices, one amino (N)-terminus, and one carboxyl (C)-terminus.^77^ As a part of the chemokine receptor group, CXCR4 plays a vital role in various physiological functions and signaling mechanisms. CXCR4 is a member of the chemokinereceptors and plays a role in multiple signaling pathways and biological functions. Protein kinases are triggered and intracellular Ca^2+^ mobilization is facilitated by chemokine receptors, which convey the effects of chemokines in target cells.^78^ CXCR4, the chemokine receptor for SDF-1 (CXCL12), plays an essential role in preserving hematopoietic stem cells (HSCs) in the bone marrow microenvironment.^79^ Consequently, the FDA-endorsed CXCR4 blocker, AMD3100 (AMD) (Mozobil), frequently used for HSC mobilization before bone marrow transplants, substantially affects bone marrow activity.^80^ It is essential to note that CXCR4 is specifically overexpressed in certain MM cells, with 60% of patients exhibiting elevated CXCR4 levels.^81^ Although earlier research has investigated the combined impacts of AMD and BTZ in MM treatment^82^ and the possibilities of liposomal drug delivery systems that target CXCR4 antagonists.^83^ C–X–C motif chemokine receptor 4 (CXCR4) is significantly upregulated in various cancers, including those of the breast, prostate, lung, and colon, and may further facilitate tumor progression and metastasis.^84^

The phospholipid framework on the liposome's surface was connected to the CXCR4 antagonist (AMD) through a carbodiimide-mediated nucleophilic substitution cross-linking method. Initially, the liposome blend was combined with 12 mg mL^−1^ of *N*-(3-dimethylaminopropyl)-*N*′-ethyl carbodiimide hydrochloride (EDC) and 5.5 mg mL^−1^ of *N*-hydroxy using an orbital shaker operating at 700 rpm. sodium sulfosuccinimide in phosphate-buffered saline (pH = 7).^32^

### 6.3. Hyaluronan

The naturally occurring polysaccharide hyaluronic acid (HA) is abundant in the human body and is essential for various physiological functions.^85^ A fundamental component of the extracellular matrix, HA is found in various tissues and structures, mainly in the eye, joints, connective tissues, and the skin. The significant hydrophilic nature of HA provides it with unique properties, such as the ability to form thick gel-like formations, retain moisture, lubricate joints, improve skin firmness and elasticity, hydrate tissues, and play a vital role in the healing of damaged tissues.^86^ The glycosaminoglycan hyaluronic acid consists of recurring dimers of the disaccharide glucuronic acid and *N*-acetylglucosamine. Unlike other glycosaminoglycans, this polysaccharide is unique because it does not contain sulfated groups and is not linked to protein structures. CD44, RHAMM (receptor for hyaluronic acid-mediated motility), LYVE1 (lymphatic vascular endothelial hyaluronan receptor 1), LAYN, hyaluronan receptor for endocytosis (HARE/stabilin-2), and toll-like receptors (TLRs) are some of the receptors in which HA associates.^87^ Numerous genes linked to cell survival and growth are expressed at higher levels when HA binds to the CD44 receptor, triggering crucial intracellular signaling pathways. The rearrangement of the actin cytoskeleton caused by this interaction leads to active cell migration.^88^

### 6.4. CD38 and CD138

**Fig. 7:**
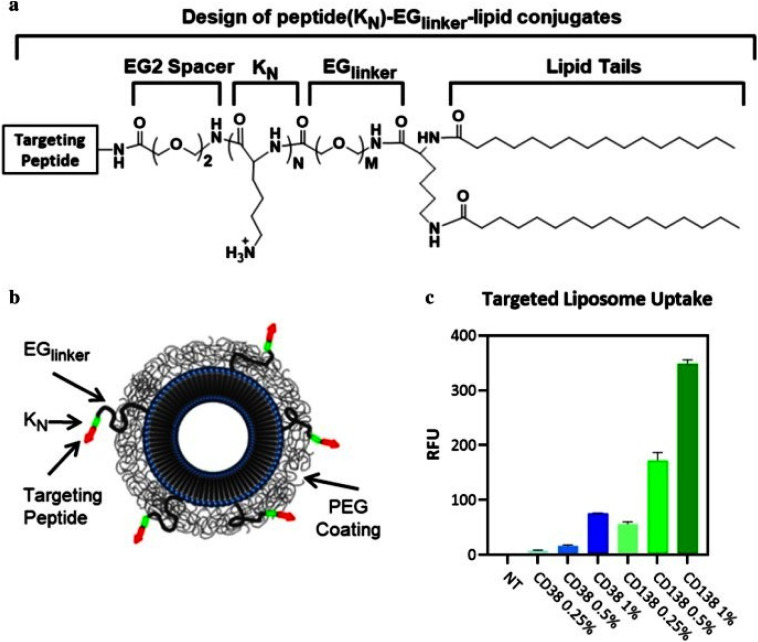
(a) Structure of liposomal nanoparticles linked with peptides and their uptake by H929 myeloma cells at designated targets. Different oligolysine (KN) quantities and EG peptide–linker lengths are incorporated in the creation of the peptide (KN)–EGlinker–lipid conjugates. (b) Illustration of the nanoparticles that home in on peptides. (c) Absorption of nanoparticles that focus on CD38pep or CD138pep at varying concentrations. Adapted with permission under a Creative Commons CC BY License from ref. 89. Copyright 2020 Springer Nature.

Plasma cells exhibit the heparan sulphate proteoglycan CD138, which influences myeloma cell attachment and maturation, thus serving as a significant therapeutic target. Initially, a lipid blend consisting of 1,2-distearoyl-*sn*-glycero-3-phosphocholine (DSPC), PEG-modified lipids, cholesterol, and peptide–lipid conjugates was created using chloroform to fabricate the liposomal nanostructures. Subsequently, a lipid film was produced by evaporating the mixture, further hydrating it with phosphate buffer solution (PBS), and extruding it through a 0.05 µm polycarbonate membrane to yield uniform nanoparticles. CD38 and CD138 peptides were then separately conjugated to the surface of the liposomes using oligolysine sequences and ethylene glycol spacers to ensure stability and specificity. DOX was encapsulated in both formulations *via* pH-sensitive linkers designed to release the drug upon cellular uptake in acidic intracellular environments. *In vivo*, CD38-targeted nanoparticles showed significantly increased uptake by tumor cells and enhanced effectiveness compared to CD138-targeted nanoparticles, which was not expected based solely on the *in vitro* findings (Fig. 7).^89^

The creation of specialized liposomal nanoparticles aimed specifically at targeting MM was achieved by utilizing anti-CD138 functionalization. The functionalized liposomal nanoparticles enabled the administration of mertansine at eight times the dosage of the equivalent free medication while remaining under the maximum tolerated dose. Compared to the control group, the *in vivo* results additionally indicated that the nanoparticles provided nearly total suppression of tumor growth, with an estimated 99 percent inhibition by day 10 and no noticeable systemic harm.^40^

### 6.5. B-cell maturation antigen

Delayed memory B cells and plasma cells express B-cell maturation antigen (BCMA), which is a transmembrane glycoprotein that is part of the tumor necrosis factor receptor family.^90^ BCMA is crucial for preserving the lifespan of plasma cells when paired with the ligands B cell-activating factor (BAFF) and proliferation-inducing ligand (APRIL).^91^ Recently, multiple therapeutic strategies have been developed to target BCMA, including bispecific antibodies, antibody–drug conjugates (ADCs), and chimeric antigen receptor (CAR) T-cell therapies, establishing BCMA as an important therapeutic target in MM.^92^ In this case, B-cell maturation antigen (BCMA) acts as a key target antigen for myeloma immunotherapy because it is predominantly expressed on cancerous plasma cells and not found in other tissues, making it a suitable choice for immunotherapeutic approaches in MM compared to CD38 and signaling lymphocytic activation molecule family 7 (SLAMF7).^93^ Currently, HDP-101 has attracted considerable interest as an anti-BCMA antibody due to promising results in early-stage clinical studies. It was stated that HDP-101-conjugated liposomes could be used as a therapeutic strategy for the treatment of multiple myeloma.^94^

## 7. Dual targeting therapies

**Fig. 8:**
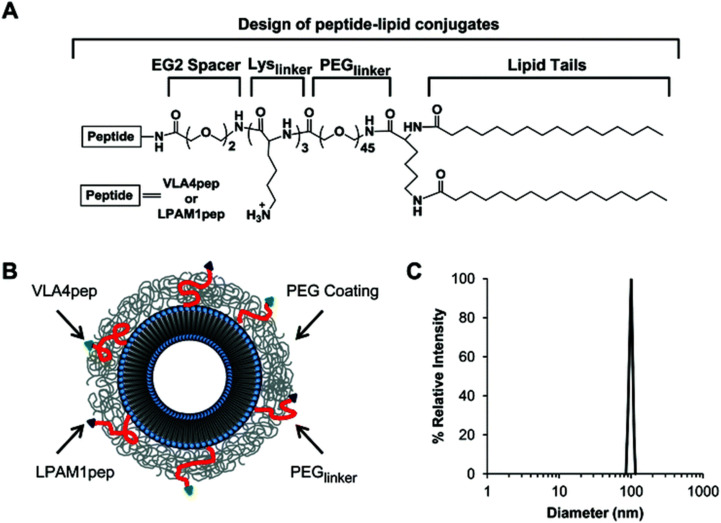
(A) The structure of the peptide–lipid conjugate. (B) Schematic representation of the dual peptide-targeted liposome. (C) DLS analysis of the liposome. Adapted with permission from ref. 98. Copyright 2019 Royal Society of Chemistry.

The presence of Very Late Antigen-4 (VLA-4, known as α4β1 integrin) and Leukocyte Peyer's Patch Adhesion Molecule-1 (LPAM-1, referred to as α4β7 integrin) in MM is often associated with unfavorable survival rates due to the development of drug resistance, rendering them as appealing targets for treatment.^95^ However, the extensive presence of these integrins in normal tissue limits the therapeutic success of methods that focus on targeting each receptor separately.^96^ As a result, a strategy aimed at two receptors enhances cell targeting and precision compared to a strategy that focuses solely on one receptor.^97^ Bilgicer and colleagues reported the design of peptide–lipid conjugates comprising: (i) VLA4pep or LPAM1pep; (ii) an EG2 spacer; (iii) a short oligolysine chain of three lysine residues (Lyslinker) to enhance peptide solubility and accessibility; (iv) a PEG2000 linker to present the targeting peptide above the PEG layer; and (v) two hydrophobic fatty acid chains for insertion into the liposomal lipid bilayer. To assess specificity, competitive binding assays were performed on NCI-H929 (V+/L+) and MM.1S (V+/L+) cells using fluorescein-labeled VLA4pep in the presence of excess unlabeled LPAM1pep. The results demonstrated that unlabeled LPAM1pep did not inhibit the binding of fluorescein-labeled VLA4pep to the cells, confirming the absence of cross-reactivity between the two peptides (Fig. 8).^98^

The folic acid-folate receptor and antibody–antigen interactions also serve as two instances of receptor–ligand pairings exhibiting strong binding affinities (*K*_d_ ≈ low nM) that have been effectively shown to be nanoparticles targeting dual receptors.^99^ As a substitute, employing peptides as the targeting ligands in a dual-receptor targeting strategy could enhance selectivity by promoting multiple low to moderate–affinity interactions, given their diverse range of affinities.^100^ Importantly, liposomal nanoparticles offer enhanced support for the multivalent display of peptide ligands, enabling precise adjustment of specific peptide loading densities to regulate binding strength and satisfy selectivity.^101^

## 8. Conclusion and future perspectives

Targeting primary MM tumors and metastatic lesions remains a major challenge in oncology due to the complex vascular architecture of the bone marrow, the presence of cancer stem/initiating cells, and the protective resistance conferred by the bone marrow microenvironment. As a targeted drug delivery platform, liposomal technology plays a critical role in the safe and highly effective treatment of malignancies residing within the bone marrow. Enhancing the *in vivo* bone marrow targeting efficiency and ligand-mediated liposome modification may facilitate the development of more effective and reliable therapeutic strategies, enabling drug delivery systems to overcome the diverse biological barriers encountered when reaching diseased bone marrow sites. Furthermore, addressing therapeutic resistance through combination therapy by using two or more agents that act on distinct biological targets has shown considerable promise and can be effectively achieved using advanced liposomal formulations. Chemotherapeutic sensitivity and efficacy may be further enhanced by disrupting interactions between tumor cells and the bone marrow microenvironment by specific inhibitors. To achieve synergistic therapeutic effects *in vivo*, the reliable co-encapsulation of multiple drugs within a single bone marrow-targeted delivery system can modulate the pharmacokinetic profiles of the encapsulated agents. The amphiphilic structure of liposomes enables the encapsulation of multiple chemotherapeutic drugs and biomolecules, while the strong negative surface charge conferred by unique polymer compositions allows for the formation of a secondary layer that facilitates adsorption and functionalization with oppositely charged cargo. Moreover, the future application of naturally derived pharmaceutics encapsulated in liposomes for MM treatment represents a promising strategy, owing to the cost-effectiveness of such agents and the relative ease of their isolation and purification.

A critical challenge associated with extensively used PEGylated liposomes is the immunogenicity of the PEG unit as a result production of anti-PEG IgM antibodies, arising from interactions between B-cell receptors and the PEG backbone. Several factors influence the immunogenicity of PEG in this context, including molecular weight, grafting density, and terminal functional groups. For instance, PEGs with molecular weights of 20 000 and 30 000, when used to modify bovine serum albumin and ovalbumin (OVA), have been reported to induce significantly stronger *in vivo* anti-PEG IgM responses.^102^ Similarly, the route of administration affects PEG immunogenicity: intravenous injection is more likely to trigger a systemic immune response, whereas subcutaneous injection tends to produce localized immune reactions.^103^ The nature of the linker between PEG and the drug carrier also impacts immunogenicity. Studies have shown that both amide and succinyl linkages between PEG and asparaginase induce comparable levels of anti-PEG antibody production following administration.^104^ To mitigate PEG-associated immunogenicity, various strategies have recently been explored. For example, modifying the linearity of PEG chains or combining PEG chains of different molecular weights (*e.g.*, carboxy-PEG2000 with methoxy-PEG550) can reduce immune recognition, while selective cleavage of PEG chains has been shown to enhance circulation time and therapeutic efficacy.^105^ PEG's antifouling properties are critical for minimizing immunological recognition, as they reduce nanoparticle interactions with cells and tissues, thereby affecting cellular uptake and transfection efficiency.^106^ Advanced approaches, such as atom transfer radical polymerization, have been used to generate libraries of PEG-lipids with varied PEG structures, charges, and molecular weights. Notably, brush-shaped poly(ethylene glycol)-methyl ether methacrylate–lipid conjugates demonstrated high transfection efficiency while effectively preventing anti-PEG antibody binding.^106^

Finally, it is important to note that liposomal delivery systems have demonstrated *in vivo* biocompatibility and biodegradability and have already received FDA approval for clinical use, highlighting the potential of next-generation liposomal nanoparticles for translational and therapeutic applications.

## Conflicts of interest

There are no conflicts to declare.

## Data Availability

No primary research results, software or code have been included and no new data were generated or analysed as part of this review.
